# Phytochemicals Mediated Remediation of Neurotoxicity Induced by Heavy Metals

**DOI:** 10.1155/2015/534769

**Published:** 2015-11-05

**Authors:** Vivek Kumar Gupta, Shweta Singh, Anju Agrawal, Nikhat Jamal Siddiqi, Bechan Sharma

**Affiliations:** ^1^Department of Biochemistry, University of Allahabad, Allahabad 211002, India; ^2^Department of Zoology, SNBVPG College, CSJM University, Kanpur 208001, India; ^3^Department of Biochemistry, College of Science, P.O. Box 22452, King Saud University, Riyadh 11495, Saudi Arabia

## Abstract

Almost all the environmental components including both the abiotic and biotic factors have been consistently threatened by excessive contamination of heavy metals continuously released from various sources. Different heavy metals have been reported to generate adverse effects in many ways. Heavy metals induced neurotoxicity and impairment in signalling cascade leading to cell death (apoptosis) has been indicated by several workers. On one hand, these metals are required by the cellular systems to regulate various biological functions of normal cells, while on the other their biomagnification in the cellular systems produces adverse effects. The mechanism by which the heavy metals induce neurotoxicity follows free radicals production pathway(s) specially the generation of reactive oxygen species and reactive nitrogen species. These free radicals produced in excess have been shown to create an imbalance between the oxidative and antioxidative systems leading to emergence of oxidative stress, which may cause necrosis, DNA damage, and many neurodegenerative disorders. This mini review summarizes the current knowledge available on the protective role of varied natural products isolated from different herbs/plants in imparting protection against heavy metals (cadmium, lead, arsenic, and mercury) mediated neurotoxicity.

## 1. Introduction

The heavy metals are generally characterized as the inorganic elements having specific gravity five times of water's specific gravity. Among many heavy metals listed into the d-orbital elements of modern periodic table, arsenic, cadmium, mercury, and lead have got prime importance because of their pathophysiological significance as their bioaccumulation in living systems may cause severe damage to the vital organs, namely, nervous system and reproductive systems, gastrointestinal tract, and mucous tissues. Though the exact mechanism of their pathogenicity is not known but there are reports from various laboratories indicating that the exposure of these heavy metals or their excess accumulation in the body tissues may induce production of free radicals particularly the reactive oxygen species (ROS) and reactive nitrogen species (RNS) which finally culminate into production of oxidative stress [1, 2]. Free radicals have been implicated into DNA damage, oxidation of thiol group(s) of proteins, and lipid peroxidation [3] which is associated to onset of various diseases.

Heavy metals reach into the environment from various sources such as from the crust of Earth mining and ground water, commercial products, industrial effluents, industrial wastes, vehicle emissions, lead-acid batteries, fertilizers, paints, treated woods, plastics floating on the world's oceans, and aging water supply infrastructure, folk remedies, and contaminated foods [2]. Thus heavy metals contaminate and intoxicate soil, water, and food items (agricultural and animal products). These elements may enter human systems through breathing, food or drink intake and contacts, and so forth. Upon accumulation, they induce various adverse effects in the body systems, characterized as heavy metals mediated toxicity. The present article is an endeavour to report the current account of phytochemicals mediated protection from neurotoxicity induced by certain heavy metals in animals including humans.

## 2. Heavy Metals Influencing Cholinergic and Noncholinergic Systems

**Figure 1:**
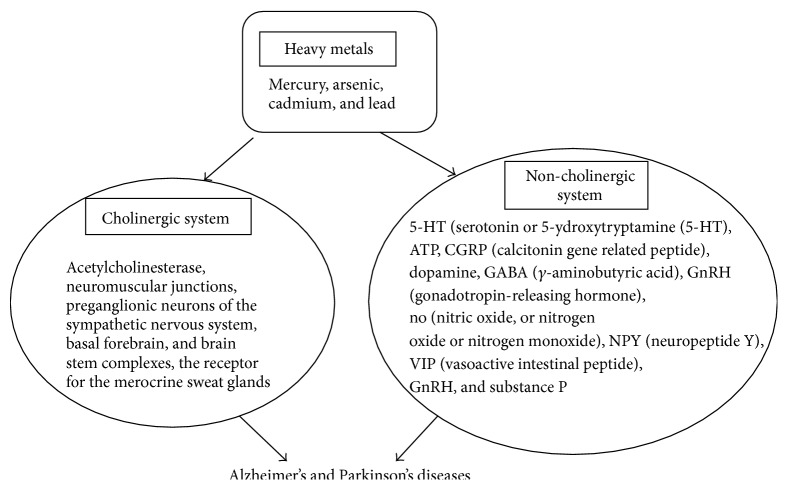
Effect of heavy metals on the cholinergic and noncholinergic systems associated to Alzheimer's and Parkinson's diseases.

Heavy metals are shown to adversely influence the functions of cholinergic and noncholinergic systems. The association of such impact of heavy metals with generation of Alzheimer's and Parkinson's diseases has been documented. A cholinergic system, the most significant modulators of neurotransmission systems in the brain, regulates physiological and cognitive functions such as memory, learning, neuronal development, and differentiation [4, 5]. Any substance or ligands having ability to produce, alter, or release acetylcholine or mimic its functions at the specific acetylcholine receptor type is categorized as a cholinergic. Similarly, any receptor or synapse using acetylcholine as a neurotransmitter is cholinergic. The parasympathetic nervous system or the preganglionic neurons of the sympathetic nervous system, neuromuscular junctions, the basal forebrain, brain stem, and the receptor for the merocrine sweat glands are also cholinergic, since acetylcholine is involved in their functions. On the other hand, the noncholinergic system involving a nonnoradrenergic, noncholinergic neurotransmitter (NANC) belongs to the autonomic nervous system (ANS) and includes serotonin or 5-hydroxytryptamine (5-HT), ATP, Calcitonin gene related peptide (CGRP), Dopamine, *γ*-aminobutyric acid (GABA), Gonadotropin-releasing hormone (GnRH), nitric oxide, or nitrogen oxide or nitrogen monoxide (NO), Neuropeptide Y (NPY), Vasoactive intestinal peptide (VIP), and substance P. The heavy metals may form complexes with these cellular factors containing oxygen, nitrogen, or sulphur and the resultant chemical species may produce toxicity which might lead to the modification in protein's structure or change in enzyme system, cellular dysfunction, and finally death of the cell. The effects of heavy metals on the cholinergic and noncholinergic systems are summarized in Figure 1.

### 2.1. Mercury (Hg)

All the natural forms of mercury (physical and chemical forms) can produce toxic effect at high doses. There are so many different forms of mercury which includes inorganic mercurous (Hg II), elemental mercury vapour (Hg), mercuric (Hg III), and organic mercuric compounds [33] which have toxic effects in organs like brain, lungs, and kidney [34]. Natural mercury is present in liquid form when its oxidation state is zero. It is categorized in transition metal element in modern periodic table and is highly reactive and toxic. The chemistry of mercury, its chemical types, their metabolic transformations in different organs, varied toxic effects in mammalian systems, and possible reaction mechanisms have been recently reviewed by Sharma et al. (2014) [2]. The toxicity of mercury is known to be mediated by its binding with the thiol group of cellular proteins and enzymes which disrupts the cellular physiology in brain and other organs. The pathophysiology of mercury toxicity relates with the exposure of an individual by all the three forms (solid, liquid, and gas) of this heavy metal. Elemental mercury crosses the blood brain barrier (BBB) readily where it concentrates in neuronal dense bodies of lysosomes and finally leads to neurotoxicity. The symptoms of mercury toxicity include gastrointestinal disorders, headache, memory loss, depression, hypertension, and neuromuscular pains in both adults and children. The therapy of mercury poisoning has been advised to be done with different compounds including 2,3-dimercapto-1-propanesulfonic acid (DMPS), D-penicillamine (DPCN), dimercaprol (BAL), and antioxidants such as N-acetylcysteine (NAC) and glutathione. The treatment of mercury intoxication is mainly managed by chelation therapy for a long period of time. However, some plant based principles have also been found to exhibit immense potential to either protect or recover from mercury induced toxicity in humans and animals. The ameliorative/protective properties of some phytochemicals have been explained in a different section and Table 1.

### 2.2. Cadmium (Cd)

Cadmium as an important member of the last d-orbital group in modern periodic table has similar chemical properties, respectively, with zinc and mercury preferably containing oxidation state +2 and low melting point. Cadmium poisoning takes place due to exposure through inhalation of cadmium fumes in the atmospheres and intake of food, water, and tobacco. Exposure to cadmium leads to occurrence of several ailments such as anaemia, osteoporosis, blood, brain, and skin related diseases. Exposure to Cd is reported to cause malfunctioning of fetus which includes ablephary, club foot, exencephaly, micrognathia, and microphthalmia. Severe exposure to calcium may harm both the fetus and the mother by enhancing the numbers of micronucleated polychromatic erythrocytes (MNPE) and micronucleated normochromatic erythrocytes (MNNE) [35]. Cadmium is known to produce free radicals and oxidative stress which is one of the mechanisms of its toxicity [36] as it combines with thiol groups of enzymes involved in antioxidant mechanisms and inactivates them. Since Cadmium can replace magnesium and calcium in certain biological systems [37], it adversely influences the concerned biological processes. Cadmium exposure may also alter DNA repair activity [38]. Cadmium intoxicated individual may suffer from neurotoxicity and respiratory, circulatory, excretory, and gastrointestinal disorders. Chronic exposure of cadmium results into appearance of certain clinical symptoms such as headache, sleep disorders and memory deficits, increased salivation, throat choking, and renal and hepatic failures, which are associated to the alterations in metabolism of neurotransmitters particularly of GABA and serotonin. Similar to mercury, the treatment of cadmium poisoning involves application of antioxidants, chelators [2], and phytochemicals as described in a separate section as well as in Table 1.

### 2.3. Arsenic (As)

Arsenic exists in trivalent arsenite (As III) and pentavalent (As V) arsenate forms. Arsenic accumulates in the body through seafood, inhalation, and absorption through skin and causes disorders in GI tract, neurotransmission, blood circulation system, and respiratory system. Arsenic exposure is known to produce free radicals and hence generates oxidative stress. It adversely influences the activities and levels of antioxidative elements such as superoxide dismutase (SOD), catalase (CAT), glutathione peroxidase (GPx), heme oxygenase-1 (HO-1), and the nonenzymatic factors such as sulfhydryl group containing peptides and proteins in human systems. Arsenic toxicity adversely influences Krebs cycle and oxidative phosphorylation leading to the depletion of energy as well as rapid depletion of thiol group containing critical peptides/proteins [39]. Arsenic poisoning is characterized by burning and numbness in the hands and feet and alterations in functions of neurotransmission and cardiovascular systems. In particular, in the patients with diabetes mellitus, arsenic toxicity may be considered as a major contributor to vascular and neurological complications [40, 41]. Normal value for urine is less than 50 mg/L and whole blood arsenic level is usually less than 1 mg/dL [39]. The treatment of arsenic intoxication is carried out mainly by chelation therapy using dimercaprol or succimer (2,3-dimercaptosuccinic acid, DMSA) and aqueous garlic extract [42]. However, some phytochemicals have been found to be very useful in combating arsenic toxicity as displayed in Table 1 and in the following section.

### 2.4. Lead (Pb)

Lead being used in batteries, metal products, and medical appliances has been found to be an important cause of concern of heavy metal toxicity in biological systems exposed to it [43]. According to the Centres for Disease Control and Prevention (CDC)-USA, a blood lead level (BLL) of 10 *μ*g/dL or above is a cause for concern. Lead mediated toxicity includes varied physiological, biochemical, and behavioural dysfunctions in human's peripheral and central nervous systems, blood circulatory, cardiovascular, excretory, metabolic, and reproductive systems [44]. It causes neurotoxicity in general but significantly reduces paediatric cognitive functions [45]. The prime mechanism of lead mediated neurotoxicity is associated with the production of excess free radical species and oxidative stress which has potential to cause perturbations in the brain [1, 46, 47]. Since Lead efficiently crosses the BBB [48] and it easily substitutes calcium ions, interferes with the regulatory action of calcium on brain cells, and disrupts its intracellular activities [48], the pathogenic symptoms of lead toxicity include hypertension, cognitive deficit [49], anaemia, peripheral motor neuropathy, and gastrointestinal disorders. The chronic lead toxicity (>70 mg/dL) in children may lead to frequent occurrence of coma, seizures, and altered mental status. The effective treatment of lead toxicity includes using preventive measures, chelation therapy, and application of some herbal preparations (Table 1).

## 3. Phytochemicals Used in Amelioration of Heavy Metals Induced Neurotoxicity

Despite the fact that the phytochemicals contain plenty of flavonoids and polyphenols like antioxidants, they may also help ameliorate the heavy metals mediated toxicity in human and other animals. The oral administration of* Arthrospira maxima* (Spirulina) has been shown to significantly reduce cadmium mediated genotoxic effects which has been corroborated to some extent with the antioxidant potential of the aforesaid algae [35]. Very recently, cadmium induced arterial and cardiac injuries could be significantly reduced by introducing dietary soybean supplementation in diet [48]. Mohammed et al. (2014) [49] have indicated that* Nigella sativa* seed oil contains strong antioxidant properties and hence may protect vital organs (brain and kidney) of the body from oxidative damages. They have proposed that* Nigella sativa* seed oil and the virgin olive oils can be used by many workers employed in cement factories wherein Cd is so frequently liberated in the environment. The phytochemicals such as vitamin C, vitamin E, phycocyanobilin, and carotenes are presented into some cyanobacterial species such as* Spirulina* and* Chlorella* which have been found to impart protection to rats exposed to lead and cadmium [26, 27, 30, 31].

The application of vitamin E has demonstrated that its administration along with cadmium caused notable reduction in accumulation of this metal in different key tissues (kidney, liver, and blood) of the body [50, 51]. Similar to the antioxidative role of fat soluble vitamin E, the water soluble vitamins (C, B1, and B6) have been found to protect the organs of rats from cadmium and lead toxicities [29, 32]. When vitamins C and E were administered together in cadmium intoxicated rats, the level of oxidative stress was sharply reduced [52, 53]. In one of the similar experiments when vitamin E was supplemented to Pb treated erythrocytes, the activity of *δ*-aminolevulinic dehydratase and lipid oxidation was significantly reduced [54]. *β*-Carotene, a red-orange pigment, fat soluble organic compound abundant in plants and fruits, has been found to serve as a precursor of vitamin A (retinol). It has been reported to be associated with increased rate of lung cancer among smokers [55].

Using garlic extract, some workers were able to demonstrate reduction in cadmium and lead mediated mitochondrial injury and apoptosis in tissue culture models as well as neural, hepatic, renal, and haematic damage in rats [56–58]. Garlic is shown to possess higher antioxidant potential than onion [59] which may be associated with the presence of allicin [7]. Aslani et al. (2010) [6] have shown that the administration of the mixture of garlic extract and dimercaptosuccinic acid (DMSA) may be able to very effectively ameliorate the rats suffering from lead poisoning. In addition to many health benefits, garlic also acts as a booster of immunity and contains antiaging substance(s) [60]. The extract of garlic has been reported to act as a potential antidote to the toxic effects of sodium arsenite [61, 62]. It is thought that the protective effect of garlic from arsenic toxicity is rendered through the thiosulfur components present in the extract which would be making some stable chemical complexes after reacting with arsenic chemical species and inactivating arsenic mediated toxicity [19]. The existing information suggests that presence of various components in the aqueous extracts of garlic in general and allicin in particular may participate in chelation of arsenic [63, 64]. The reduction in the toxicity induced by arsenic has been achieved by the coadministration of plant extracts, such as* Allium sativum*,* Aloe vera barbadensis*,* Centella asiatica*, curcumin, and* Hippophae rhamnoides* as recorded by evaluating hematological, renal, and hepatic parameters in experimental animals [19, 65–69]. In order to mitigate the toxicity of arsenic, not many plants have been explored to be used as a medicine.

However, the leaf extracts of* Annona muricata* have been found to have potential to significantly reduce arsenic mediated neurotoxicity [70–72]. The methanolic extract of the leaves of* A. muricata* was found to exhibit higher activity than the aqueous extract. They observed a dose-dependent decrease in arsenic toxicity. These authors have also indicated that tea extract could also be useful in arsenic poisoning cases to reduce the toxicity.* H. rhamnoides* is a rich source of vitamins A, C, and E; carotenoids and organic acids which gives positive effects on arsenic induced toxicity have been attributed to the presence of high content of antioxidant substances [67, 69].

The quercetin (3′,3,4′,5,7-pentahydroxyflavone), a dietary flavonoid, is found in fruits and vegetables, olive oil, red wine, and tea [18, 19]. It has been observed to contain free radical quenching activity which scavenges free radicals (Hertog et al., 1995) [18] induced by arsenic exposure when administered either alone or in combination with a thiol chelator [73, 74]. It has been observed that a nanocapsulated drug delivery system of Quercetin can provide better medication to prevent arsenic induced damage than bulk administration of Quercetin [73, 75]. The extracts from a few other traditional medicinal plants such as* Moringa oleifera, A. barbadensis,* and* C. asiatica* have also been found to offer beneficial effects by protecting the vital organs of the body probably by reducing oxidative stress [66, 68, 76] and depletion of arsenic concentration in tissue [68] and through the interactions of phytochemicals with cysteine- and methionine-rich proteins [73, 76, 77].

Tomato extract has been shown to reduce bioaccumulation of heavy metals [78] and acts as a strong antioxidant and protectant from cadmium and lead mediated toxicities in rats [79, 80]. This property of tomato may be associated to the presence of certain metal chelating proteins and phytochelatins [81]. Some Indian spices such as coriander is shown to contain phenolic acid compounds like caffeic acid, chlorogenic acid, vanillic acid, p-coumaric acid, ferulic acid (cis and trans form) [24, 25], and curcumin {1,7-bis (4-hydroxy-3-methoxyphenyl)-1,6-heptadiene-3,5-Dione} (diferuloyl methane) isolated from turmeric has anti-inflammatory and antioxidant properties [82] which enables it to protect the cadmium exposed rats from nephrotoxicity [13]. During a field trial conducted in West Bengal, curcumin was derived from turmeric [12].

For a very long time, the antioxidative properties of tea have been known [83–85]. The catechins present in green tea and the flavonoids and phenols of curry leaves as well as fruits (grapes) have been demonstrated to exhibit similar protective effects [86, 87] in the cases of lead and cadmium poisoning. The major green tea polyphenols with antioxidant properties may include (−)-epigallocatechin-3-gallate (EGCG), (−)-epicatechin-3-gallate (ECG), (−)-epigallocatechin (EGC), (−)-epicatechin (EC), (+)-gallocatechin (GC), and (+)-catechin [83, 85].

Chinthana and Ananthi (2012) [88] have reported that the neurotoxicity induced by lead in albino mice could be significantly reduced by oral administration of extract of* S. nigrum* and* S. trilobatum* leaves for 30 days. They observed substantial increase in the activities of antioxidant enzymes such as SOD, CAT, GPx, and reduced lipid peroxidation.* S. nigrum* is a weed and some species of it may be toxic to humans and animals. This plant is widely used as a traditional medicine [89]. Similar observations have been recorded by Zaidi et al. (2014) [90] when they evaluated the prophylactic or curative antioxidant efficacy of crude extract and the active constituent of* S. nigrum* leaves in the brain of stressed rat.

**Figure 2:**
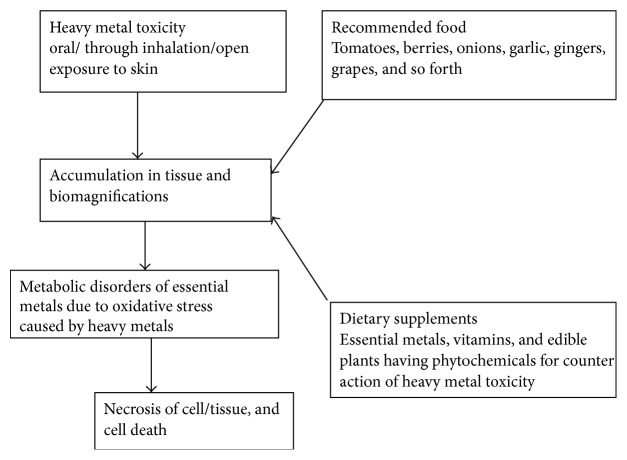
Impact of heavy metals on human system.

A summary of the key phytochemicals isolated from different plant sources and characterised for protection from heavy metals mediated neurotoxicity are presented in Table 1. A graphical sketch of protection offered by dietary supplements against the heavy metals induced alterations in biochemical and physiological functions of the body is displayed in Figure 2.

## 4. Conclusion

The heavy metals exhibit the ability to induce oxidative stress by generating ROS. Oxidative stress due to heavy metals is the outcome of a negative shift of the balance between the production of ROS and the ability of the biological systems to readily counteract the ROS mediated damage or repair of it rapidly. The heavy metals toxicity is caused either by their direct binding with thiol groups of the proteins/enzymes thereby causing perturbations in their three-dimensional conformations or by replacing the divalent metal ions from their catalytic pocket which are essentially required by concerned proteins/enzymes as cofactors for their optimal activity. In either of these situations, these biomolecules tend to lose their native characteristics due to unfolding or denaturation and then their functions are greatly compromised, which lead to serious bearings on to their biological activity and finally the cellular health. All forms of life maintain a reducing environment within their cells. This reducing environment is preserved by enzymes that maintain the reduced state through a constant input of metabolic energy. Disturbances in this normal redox state by heavy metals can cause toxic effects indirectly through the production of peroxides and free radicals that induce oxidative stress, which is responsible for the damage of several key components of the cells, including proteins, lipid, and DNA. The amelioration of neurotoxicity induced by certain heavy metals by herbal principles is of great significance as they are cost effective and highly active and lead to no side effects. In addition to them, the application of certain vitamins and a number of chelating organic molecules is being tried with the expectation that they would make coordinate complexes with these heavy metals and help remove them making the affected organs or tissues free from metal's burden and toxicity. Although coadministration of antioxidants (natural, herbal, or synthetic) or with other chelating agents has shown to improve removal of toxic metals from the system as well as clinical recoveries in animal models, still the in-depth clinical studies with preexisting or newer chelating agents are required to be done to reap real benefit with the least side effects. In case of humans, it is also important to find out the suitable dose and duration of treatment to decide the optimal therapeutic index for any of the drugs to be given in isolation or in different combinations. In conclusion, the present stage of knowledge about the impact of heavy metals in biological systems indicates the enhanced formation of free radicals and ROS or RNS or their intermediates causing neurotoxicity and other ailments and that could be efficiently managed by using different preparations from a number of traditional medicinal plants.

## Figures and Tables

**Table 1:** Phytochemicals as antidotes to heavy metals induced toxicity.

Phytochemicals	Sources	Protective functions	References
Allicin	Garlic	Reduces arsenic induced oxidative and arsenic toxicity by complex formation.	[6, 7]

Anthocyanin/flavonoids	Cherry, grapes, and berries	Anthocyanin protects against Cd-induced oxidative stress. Anthocyanin appears to effectively diminish Pb induced oxidative stress.	[8, 9]

Catechins	Tea, cocoa, peach, and berries	Catechin inhibits Cd absorption and normalises bone metabolic disorders through the bone mineral density, bone mineral content, and bone calcium content. Catechin protects hepatic cell membrane fluidity, increases cell viability, and modulates oxidative stress.	[10, 11]

Curcumin	Turmeric	Curcumin protects against Cd-induced lipid peroxidation. Curcumin binds Pb to form an excretable complex, reducing neurotoxicity and nephrotoxicity.	[12–15]

Naringenin	Orange, grapefruit, and tomato	Naringenin quenches free radicals, recovers antioxidant enzyme activity, and chelates Cd.	[16]

*γ*-Oryzanol	Rice	*γ*-Oryzanol reduces the testicular Cd concentration, improves *δ*-aminolevulinic acid dehydratase (ALAD) activity, and prevents lipid peroxidation.	[17]

Quercetin	Onion, tomato and radish olive oil, red wine, tea, and so forth	Induces the expression of endothelial nitric oxide synthase (eNOS), inducible nitric oxide synthase (iNOS), and cyclooxygenase-2 (COX-2). Quercetin modulates the mitogen-activated protein kinases (MAPKs) and nuclear factor kappa B (NF-*κ*B) signalling pathway and forms excretable complex with Pb hydroxyl and superoxide groups scavenge radicals, whereas the phenolic groups act as possible chelating sites.	[18–23]

Phenolics	Coriander, fruits, vegetables, and tea	Act as antioxidants.	[13, 24, 25]

Phycocyanobilin	Cyanobacteria (*Spirulina* and *Chlorella*)	Probably acting as antioxidants.	[26, 27]

Puerarin	*Pueraria mirifica* plant	Puerarin modulates the phosphoinositide-3-kinase (PI3K)/protein kinase B (Akt)/endothelial nitric oxide synthase (eNOS) pathway, reduces reactive oxygen species, and protects against DNA damage and apoptosis.	[22, 28]

Vitamins A (*β*- carotenes), B1, B6, C, and E	*Nigella sativa*	Act as antioxidants.	[29–32]
